# An action research approach to facilitating the adoption of a foot health assessment tool in India

**DOI:** 10.1186/s13047-015-0108-3

**Published:** 2015-09-16

**Authors:** Michael Harrison-Blount, Michelle Cullen, Christopher J. Nester, Anita E. Williams

**Affiliations:** School of Health Sciences, College of Health and Social Care, University of Salford, Frederick Road, Salford, M6 6PU UK

**Keywords:** Diabetes, Action research, Foot health assessment

## Abstract

**Background:**

India has a diabetes population that is growing and alongside this, the incidence of limb threatening foot problems is increasing. Foot health care provision does not yet meet this demand. In one locality in India, clinicians had an unstructured approach to foot health assessments resulting in poor adoption of evidence based guidelines from the West and a persistence of serious foot complications. There was the perception that existing assessment tools did not take into account the local cultural, organizational and professional needs and there was a lack of ownership of any potential solution to the problem. Therefore, the aim of this work was to facilitate the ownership and development of a foot health assessment tool for use in the Indian context. In order to achieve this an action research approach was chosen.

**Methods:**

Participants were facilitated through the action and implementation phases of the action research cycle by the researchers. The action phase included generating a list of potential items for inclusion in the tool from a review of the literature to provide an evidence based foundation for the foot health assessment tool. A modified Delphi method was used to further refine the contents of the tool. Members of the Delphi Panel (*n* = 8) were experts in their field of medicine and experts in delivering health care within services in India.

**Results:**

The outcome of the study was the adoption of a locally developed foot health assessment tool (Salford Indian Foot Health Assessment Tool, SIFT). It contains thirteen sections, which reflect the risk factors identified for assessing foot health agreed by the participants to fit the Indian context. The SIFT is supported with evidence based guidelines from the West and a training program was delivered by the researchers in order to support its implementation into clinical practice.

**Conclusion:**

An action research approach has facilitated the development and implementation of a locally created and owned foot health assessment tool. This in turn has resulted in the integration of evidence-based guidelines from the West with consideration to local cultural, organizational and professional needs and ultimately the needs of their patients. Further work is underway evaluating the outcomes of the SIFT in practice.

**Electronic supplementary material:**

The online version of this article (doi:10.1186/s13047-015-0108-3) contains supplementary material, which is available to authorized users.

## Background

India is second only to China in terms of the size of its diabetes population (65.1 million and 98 million respectively [1]), and incidence is 5.73 % higher than in Europe. The complications of diabetes for the foot are preventable but can only be achieved if the complications are identified early enough and strategies provided in a timely way. With the Indian diabetes population growing it is important to support development of foot health care services by transferring evidence based guidelines from those health care contexts with established experience. However, for this to be effective and have lasting effect, the adoption of best practice must reflect health and social contexts within India and tailored to meet the needs of the local patients and organizations.

Evidence based assessment tools for the identification of foot health complications and their associated risk factors should be the cornerstone of an effective strategy for diabetes foot care in India. However, there are multiple barriers to the implementation of these assessments. It is known that there are insufficient treatment facilities, a lack of structured education programmes and there are cultural and religious influences on behaviors’ related to health. These factors can often explain the late presentation of foot ulcers, walking barefoot, poor footwear, traditional healing techniques and dangerous aesthetic foot treatments [2].

Effective implementation of change in foot care services, such as the use of new assessment tools, requires a tailored approach that is sensitive to the local factors affecting change. Previous work has identified the need to use different approaches, methods and therapeutic strategies in the Eastern (or Indian) context to achieve the goal of transferring evidence based guidelines [3–5]. Furthermore research has explored the need for understanding the values required to successfully embed evidence based practice [6–8]. These values include a clear understanding of the skills and awareness of the person adopting the evidence based practice (the adopter) and the need for ensuring that the research is accessible (the innovation) and matches professional consensus (the organization) [6]. Furthermore that any education provision to support the embedding of evidence based practice must involve active participation of the adopter, with the ultimate aim of increasing knowledge and changing professional behavior [6–8]. Thus, the process through which evidence based guidelines are transferred must embrace these values.

Several authors [6, 9, 10] advise that the characteristics of the specific context, the new knowledge, the clinicians involved and their possible interactions need to be taken into account when implementing change. An action research approach embraces these points and is carried out in collaboration “with” stakeholders within their natural context rather than them being subjects ‘in’ research.

In the first paper of this series [5] researchers used an action research approach to illustrate the importance of process and engagement in driving change and the adoption of evidence based practices by those associated with foot health management in India. The work utilized the problem identification and action-planning phases of the action research process [5] in order to support change in foot care in one locality in India. This was successful in achieving local ownership and identification of the clinicians own problems and, specifically, a desire to develop and implement a foot health screening/assessment tool as a potential solution.

This paper is the second part of the action research approach and uses the need for an agreed foot health assessment tool as an opportunity to facilitate local ownership of health care solutions which have evidence based practices embedded. We use the phases of action, reflection and evaluation required to complete the action research cycle. The aim of this work was to drive consensus among local health care professionals in the details of the foot assessment tool in order to create a locally agreed and owned tool for assessment of foot health status in the Indian context. Aligned with the development of the tool was the need to ensure the adoption of evidence based guidelines, whilst being aware of cultural, organizational and professional needs.

## Methods

Approval for the study was obtained from the University of Salford Research, Innovation and Academic Engagement Ethical Approval Panel (Approval number HSCR12-22) and the hospital governance team at a major university hospital situated in Chennai, India.

An action research approach was adopted to ensure that the assessment tool was based on evidence based guidelines [11], whilst being culturally sensitive and applicable to local need. The foundation phase of the action research cycle has been achieved [5] and the focus of this paper is the action, reflection and evaluation phases.

Within the framework of action research processes, a consensus building approach (a modified Delphi technique) was used to facilitate definition of the foot health assessment tool. The participants were facilitated through an iterative process that used five rounds of questions, data collection and analysis techniques interspersed with feedback. It was a process through which ownership was given to the clinicians, with the results being used to define the components of a draft assessment tool that was then checked for face and construct validity. Several authors recommend a staged approach to developing an assessment tool and this was utilized during the process, [12–14].

### Stage 1 – Preliminary conceptual decisions

To guide the development process, a list of prerequisites was created from the results of the action planning phase of the action research cycle in which the clinicians had participated and is reported in a previous paper [5]. This list of prerequisites highlights the standards that the participants feel any subsequent tool should achieve. The list can be viewed in the results section.

### Stage 2 – Initial category and Item generation

A list of items were presented to participants in Round 1, relating to factors associated with diabetes foot complications, and these were generated from a focused search of the literature that used a thematic framework approach to identify content cross referenced with a review of clinical guidelines. The literature search identified 392 articles, of which 295 were excluded. Sixty one further articles were excluded because they were duplicates, did not report foot related complaints, reported on screening/assessment tools for non-foot related conditions or reported complaints or risk factors not highlighted in best practice guidelines [11]. Thirty-six studies were included in the final qualitative synthesis and were cross-referenced with national and local UK guidelines. This thematic analysis produced a list of 20 risk factors and 40 screening tests/checks for possible inclusion in the assessment tool.

### Stage 3 – Assessment of face and content validity

In the context of the diabetic foot, validity refers to the degree to which the assessment measures the risk factors presenting in the lower limb. Face validity is the lowest level of validity and based upon the personal opinions of the observer. Content validity is determined by theoretical reasoning that a foot health assessment tool adequately measures selected foot health variables. When a foot health assessment tool is believed to include the domains that are required to adequately assess the foot its content is considered valid (content validity). We achieved consensus on the items to be included in the tool as well as the phrasing of such items and this process also enhanced the ownership of the assessment tool.

### Stage 4 – Field trials to assess consistency and construct validity

The assessment tool created by the clinicians (Salford Indian Foot Health Assessment Tool (SIFT)) was piloted to begin the process of assessing consistency, to achieve construct validity and to allow clinicians to test local implementation of their own solution. This process will continue following completion of the Delphi and will be reported in a follow up paper.

**Fig. 1:**
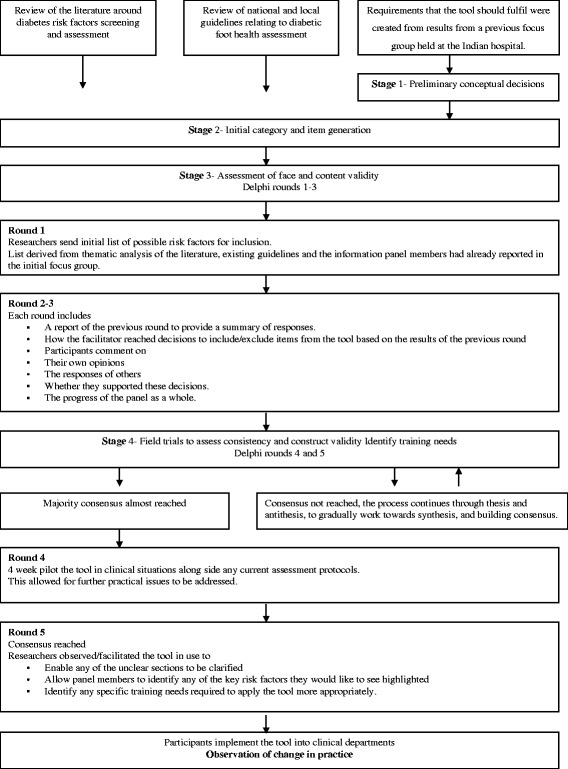
The Delphi procedure-overview of the process

An overview of the Delphi process can be seen in Fig. 1.

### Selection of Delphi panel members

The authors recruited to the Delphi panel those participants who had taken part in the first phase of the action research model [5], as it would be these clinicians who would be embedding the evidence base into clinical practice. This provided continuity through the subsequent action and implementation phases. Although not experts in the assessment and triage of foot health, this was not necessary, since they were all experts within their chosen disciplines and in the Indian health care context. This knowledge was the foundation of their role in the Delphi process, since what is ‘evidence based’ in clinical terms was established through the literature and existing guidelines search in the initial category and item generation phases.

The term item is used to mean both the risk factors and tests/assessments used. Previous authors have stated that consensus is reached when a positive response level of 75 % or greater from the participants is reached [12–17].

### Round 1

Information about the aim of their participation, the methods used and invitation to be part of the change process was sent to the participants. The researchers sent the initial list of possible risk factors for inclusion in the foot health assessment tool to the participants. This was the list derived from the initial literature search and the information participants had already reported as list of criteria they would deem important in a foot assessment tool from a previous focus group [5]. This provided early ownership of the knowledge to be discussed, free from the influence of peers and the dynamics of group discussions. The aim was to collect information on each participant’s opinion regarding the importance of each risk factor. To help the decision making process the authors also included a summary of the evidence that supported each risk factor. Participants were asked to rate the importance of inclusion of each risk factor using a Likert scale (5 being essential to *include* and 1 being essential to *remove*). The authors also gave participants the opportunity to comment on any of the risk factors included in the tool. Responses were accumulated to devise an agreed criteria list, which was then sent out for comments and amendments in round 2.

### Round 2

The researchers used the results of round 1 to select risk factors for which there were high levels of agreement for inclusion/exclusion from the final tool. Risk factors that rated as 5 and 4 by at least 75 % (6 participants) were included. Risk factors rated, as 3 by at least 75 % of the members were included in this round to allow for re consideration and those rated as 2 and 1 were excluded.

For round 2 the participants were asked to review the comments or amendments made to the risk factors in round 1 and re-evaluate any of those risks. At this stage panel members were provided with the tests/assessments to be used to evaluate each risk factor and again asked to rate their inclusions on a Likert scale (5 being essential to include and 1 to be removed). Again panel members were given the opportunity to provide comments and additionally participants were asked: Would you like to see a number of key Risk factors highlighted on the final assessment tool? And do you endorse the Delphi procedure so far? If no please give details of the aspects of the procedure which you do not support and list any suggestions you have for improvement. This latter question challenged participants to acknowledge their participation in a group exercise and reaffirm their belief in its progress. This was important to bolster confidence in the process in which they were engaged and to validate their contributions as ‘valued’.

### Round 3

The results of round 2 were used to select the final risk factors, tests and assessments for inclusion in the final tool. Risk factors rated 3 at round 2 that had been re-rated as 5 and 4 by at least 75 % of the participants who replied in this round were selected for inclusion in the final tool. Items that remained rated as 3 or had been re-rated as 2 or 1 were excluded.

Tests/assessments that rated as 5 and 4 by at least 75 % of the participants who replied in this round were selected for inclusion in the tool. Tests/assessments rated, as 3 by at least 75 % of the members were included for the next round to allow for re consideration and those rated as 2 and 1 were excluded.

This third round asked the panelists to review the inclusion and exclusion results for both items and to indicate whether they thought that an item should be included or excluded. Participants were also asked to comment on any item that they would like to rephrase or whether they wished to add an item to the tool and to provide suggestions of such changes.

### Round 4 (Pilot)

The results of round 3 were used to select any rephrased or additional items to be included in the foot health assessment tool. Risk factors and tests/assessments selected for inclusion at this stage were presented to the participants for them to indicate whether they agreed with the proposed rephrasing or additional items, and if not to suggest alternatives. Risk factors and tests/assessments that rated as 5 and 4 by at least 75 % of the participants who replied in this round were selected for inclusion in the tool. Risk factors and tests/assessments rated as 3 by at least 75 % of the members were included for the next round to allow for re consideration and those rated as 2 and 1 were excluded.

This provided a new level of contribution by participants, such as rewording specific items, and thus provided ownership of the precise details and language used.

The clinicians who had been part of the process were asked to pilot the tool over a 4-week period on patients presenting with foot health problems within their own specific disciplines. This brought to life their own efforts in practical terms, in front of their colleagues and patients, and thus reinforced the productivity of the exercise in which they had participated. They were asked to consider, the practicalities of the risk factors and tests/assessments for suggested inclusion in the final tool, how the test and assessments would be recorded, any further additions they may want to make to the tool and to suggest solutions and alternatives to any comments made. Participants were asked to record their decision using either yes, no or unclear with consensus reached at a level of 75 % agreement as with previous rounds. Additionally participants were asked to record any further items they would like to have considered for inclusion in the tool following the pilot. These were recorded as a list and distributed to the participants.

### Round 5 (Visit) modification of the Delphi procedure

This modification allowed the researchers to see the tool being used in a clinical setting in order to influence the development stages thereby improving face validity and overcoming the potential interpretation difficulties. The results of round 4 were used to select the content of the final foot health assessment tool. All current and additional items rated as ‘yes’ by at least 75 % of the participants who replied in this round were selected for inclusion in the final tool. All current and additional items rated as ‘no’ by at least 75 % of the participants who replied in this round were excluded. We considered responses regarding items marked as ‘unclear’ by at least 75 % and asked the respondents to revisit the guidelines for clarification.

In round 5 the final version of the Salford Indian Foot Health Assessment Tool (SIFT) and the guidelines were presented to the panel members for use. The researchers spent one week with participants in order to provide support and to identify any specific training needs.

## Results

Eight of the eleven clinicians invited to take part consented to participate in the Delphi procedure. The specialties of the clinicians are listed in Table 1.

**Table 1:** Participants involvement and clinical specialties

Participant (P) code	Role	Agreement to take part in the process
P1	Consultant General Physician (Head of service)	Agreed
P2	Consultant General Physician	Requested not to take part
P3	Consultant Dermatologist	Agreed
P4	Orthopaedic surgeon (Lead member of staff, key contact)	Agreed
P5	General surgeon (Head of service)	Agreed
P6	Consultant Sports Medicine	Agreed
P7	Consultant Diabetologist (Lead member of staff, key contact)	Agreed
P8	Head of Physiotherapy	Agreed
P9	Head orthotist	Agreed
P10	Consultant Vascular Surgeon (Head of service)	Requested not to take part
P11	Consultant endocrinologist (Head of service)	Requested not to take part

### Preliminary conceptual decisions

**Fig. 2:**
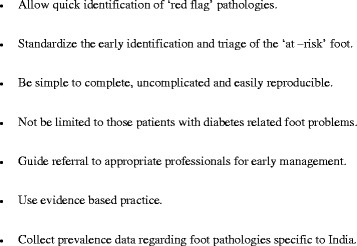
List of prerequisites to be met by a foot health assessment tool designed for this locality

The researchers and participants of the focus group in the problem identification and action planning stages decided that the foot health assessment tool should have the following prerequisites (Fig. 2).

### Delphi rounds 1–3: Assessment of face and content validity

The result of this study was the design of a foot health assessment tool (SIFT) developed by those who would use it in clinical practice. This was achieved using a modified Delphi method embedded in an action research process, which aims to facilitate change through ownership.

### Delphi Round 1

All eight of the participants who agreed to take part in the procedure returned completed questionnaires. Following the results of this round, ten risk factors were selected for inclusion, eight risk factors were removed from the tool as they were rerated at 1 or 2 on the likert scale by >75 % of the participants and 2 risk factors were put forward to be rerated as part of round 2 (Table 2).

**Table 2:** Delphi results: Round 1

Original 20 risk factors identified for possible inclusion	% expert acceptance	Likert rating	Feedback put forward for round 2
Infection	100	4-5	To be included
Ischemia	100		
Neuropathy	88		
Ulceration current	88		
Deformity	88		
Dermatological	75		
Amputation	88		
Smoking	100		
Footwear	88		
Charcot	88		
Vascular surgery	88	3	To be rerated
Ulceration previous	88		
Trauma	75	1–2	To be excluded from process
Education	88		
Age socioeconomic	100		
Plantar pressures	88		
Self care	88		
Retinopathy/visual impairment	100		
Nephropathy	88		
Glycaemia	88		

### Delphi round 2

Of the eight practitioners involved with the process seven returned completed questionnaires. The eighth member of the panel had reported being too busy this time to take part, but still wished to be part of further rounds.

Following the results of this round, the ten risk factors selected for inclusion were not challenged and would therefore be included in the final tool. The eight risk factors removed from the tool were all agreed and therefore excluded from the process. The two risk factors where consensus had not been reached had been rerated, one to be included in the final tool and the other to be amended, and then added to the final tool (Table 3).

**Table 3:** Delphi results: Round 2 Consensus for risk factors and tests/assessments

Round 2 consensus for risk factors				Round 2 consensus for tests/assessments			
Risk factors confirmed for inclusion in the final tool	% expert acceptance	Likert rating	Comments or amendments on previous round	Original list of Tests/assessments for possible inclusion	% expert acceptance	Likert rating	Feedback put forward for round 3
Infection	100	4–5	Not challenged	Visual assessment of Redness, warmth, Pus, odor, swelling	100	4–5	To be included
Ischemia	100			DP/TP pulses	100	4–5	To be included
				Temp touch	86		
				ABPI	86	1–2	To be excluded
				Doppler	86		
				CRT	86		
				TBI	100		
				Duplex	100		
				TcPO2	100		
Neuropathy	100			Monofilament	100	4–5	To be included
				Tuning fork	100		
				Vibratip	100	1–2	To be excluded
				Ipswitch touch test	100		
				Ankle reflex	100		
				Neurotip	86		
				Biothesiometer	100		
Ulceration current	100			Yes/no	100	4–5	To be included
Deformity	100			Charcot	100	4–5	To be included
				Claw/hammer toes	100	3	To be rerated
				HAV	86		
				Flat foot	100		
				Prom Met heads	100		
Dermatological	100			Corns and Callus	86	4–5	To be included
				Dry	86		
				Cracked	100		
				Fungal	86		
				Blisters	100		
				Extravasation tissue	86		
				Maceration	86		
				Nail problems	86	3	To be rerated
				Atrophy	86	1–2	To be excluded
Amputation	100			Yes/no	100	4–5	To be included
Smoking	100			Yes/no	100	4–5	To be included
Footwear	100			Style	100	4–5	To be included
				Toe box	100	3	To be rerated
				Wear	100		
				Insoles	100		
				Fit	100		
				Condition	86		
Charcot	100			Visual assessment of redness, unilateral temp difference, swelling	100	4–5	To be included
Vascular surgery	86	4–5	Vascular surgery altered to Significant surgical history	Yes/no	100	4–5	To be included
Ulceration previous	86		Included	Yes/no	100	4–5	To be included
Trauma	86	1–2	Not challenged				
Education	86						
Age socioeconomic	100						
Plantar pressures	86						
Self care	86						
Retinopathy/visual impairment	100						
Nephropathy	86						
Glycaemia	86						

The results following this round found that 5 risk factors would be assessed by a yes or no option, fifteen further tests/methods of assessment were selected for inclusion, fourteen tests/assessment methods were removed from the tool, including two that had not been sent to participants as the risk factor they assessed had already been excluded in a previous round. Ten tests/assessment methods were put forward to be rerated in round 3 (Table 3).

In this round six of the seven participants were in favor of seeing a number of key risk factors highlighted on the final assessment tool. The member of the panel who stated they were ‘unclear’ about highlighting certain risk factors went on to explain that they felt this could only be done if ’it did not over complicate the tool and make it difficult for all staff to use and make sense of’ as the majority of members were in favor of this, the researchers decided to investigate highlighting the risk factors in round five during observation of its use in practice. At this stage all seven of the panel members reported that they endorsed the Delphi procedure so far and were happy to continue with the process.

### Delphi round 3

All eight of the practitioners involved with the process returned completed questionnaires.

Following the results of this round the twenty tests/assessment methods selected for inclusion were not challenged and would therefore is included in the final tool. The twelve tests/assessment methods removed from the tool were all agreed and therefore excluded from the process. Of the ten tests/assessment methods where consensus had not been reached and had been rerated, one was included into the final assessment tool and the remaining nine were excluded from the process (Table 4).

**Table 4:** Delphi results: Round 3

Original 20 tests/assessments identified for possible inclusion	% expert acceptance	Likert rating	Comments or amendments on previous round
Infection - Visual assessment	100	4–5	Not challenged and therefore included
DP/TP pulses	100		
Temp touch	100		
Monofilament	100		
Tuning fork	100		
Ulceration current – yes/no	100		
Ulceration previous – yes no	100		
Deformity – Charcot	100		
Corns and Callus	100		
Dry	100		
Cracked	100		
Fungal	100		
Blisters	100		
Extravasation tissue	100		
Maceration	100		
Amputation – yes/no	100		
Smoking – yes/no	100		
Footwear - style	100		
Charcot - Visual assessment	100		
Significant Surgical History– yes/no	100		
Nail problems	88	4–5	Included
ABPI	88	1–2	Not challenged and therefore excluded from the process
Doppler	75		
CRT	88		
TBI	100		
Duplex	100		
TcPO2	100		
Vibratip	100		
Ipswitch touch test	100		
Ankle reflex	100		
Neurotip	88		
Biothesiometer	100		
Atrophy	100		

The additional results in this round report those items that the panel wished to rephrase or add. During this round no additional risk factors were added, four risk factors were rephrased, one was subdivided into two, and four were categorized under one of the other existing risk factors and therefore were moved into the tests/assessment methods section. In addition twelve further test/methods of assessment were added, two of which had further subdivisions, four were rephrased and three were subdivided (Table 5).

**Table 5:** Delphi results: Round 3

Risk factors put forward for rephrasing or subdivision	Suggested rephrasing or subdivision	Tests/assessments put forward for rephrasing recategorizing or subdivision	Suggested rephrasing, recategorizing or subdivision	Suggested tests/assessments to be added to current list
Infection	Cellulitis	Visual assessment of Redness, warmth, Pus, odor, swelling	Yes/no Move to skin conditions	
Deformity				Amputation
				Forefoot
				Rear foot
				Other
Ischemia	Peripheral vascular assessment	DP/TP pulses	Palpable - Yes/no	Intermittent claudication
				Oedema
				Rest pain
		Temp touch	Normal /Abnormal	
Neuropathy	Neurological assessment	Monofilament	10 sites for monofilament	
		Tuning fork	Hypo pigmented skin lesions	
Ulceration current		Yes/no	Move to skin conditions	
Ulceration previous		Yes/no		
Dermatological	Skin conditions	Maceration	Hyperhydrotic	Normal
		Dry	Hypohydrotic	Interdigital rash
		Cracked	Fissure	Cellulitis
	Nail conditions			onychauxis
				onychomycosis
				paronychia
				onychocryptosis
				other
Amputation		Yes/no	Move to deformity	
Footwear		Style	Type	Habits
				Worn always
				Outdoors only
				never
				Indoors only
Charcot		Visual assessment of Redness, unilateral temp difference, swelling	Yes/no Move to deformity	

Panel members were also asked in this round if they agreed with the procedure of being asked whether they would support the instructions issued in round 4 and whether they would be willing to comment on any changes. All eight panel members agreed to this process.

### Delphi rounds 4 & 5: Field trials to assess consistency and construct validity

Field trials to assess consistency and construct validity are ongoing, as at this stage we have not undertaken any formal quantitative analysis. The first stages of the validation process have begun by piloting the tool in clinical practice.

### Delphi round 4

All eight of the practitioners involved with the process returned completed questionnaires and took part in the pilot of the tool over a four week period, on patients presenting with foot health problems within their own specific disciplines, where they were asked to consider the practicalities of the tool in a clinical setting.

Following the results of the completed questionnaires the eight risk factors chosen and where rephrased were not challenged and therefore included in the pilot tool. Of the thirty one tests/assessment methods chosen, none of the rephrased or additional items were challenged and therefore included in the pilot tool. No alternatives were suggested for any of the current items (Table 6).

**Table 6:** Delphi results – Round four consensus on rephrased items and additional tests/assessments

Round four consensus on rephrased items						Round four consensus on additional assessments/tests	
Risk factors for inclusion	Suggested risk factors to be rephrased or subdivided	% expert consensus	Tests/assessments for inclusion	Suggested tests/assessments to be rephrased or subdivided	% expert consensus	Suggested tests assessments to be added	% expert consensus
Infection	Cellulitis	100	Visual assessment of Redness, warmth, Pus, odor, swelling	Yes/no Move to skin conditions	100		
Ischemia	Peripheral vascular assessment	100	DP/TP pulses	Palpable - Yes/no	100	Intermittent claudication	100
			Temp touch	Normal/Abnormal	100	Oedema	100
						Rest pain	86
Deformity						Amputation	100
						Forefoot	75
						Rear foot	75
						Other	75
Neuropathy	Neurological assessment	100	Monofilament	10 sites for monofilament	86		
			Tuning fork	Hypo pigmented skin lesions	100		
Ulceration current			Yes/no	Move to skin conditions	86		
Ulceration previous			Yes/no				
Dermatological	Skin conditions	86	Maceration	Hyperhydrotic	100	Normal	100
			Dry	Hypohydrotic	100	Interdigital rash	75
			Cracked	Fissure	75	Cellulitis	86
	Nail conditions					onychauxis	75
						onychomycosis	75
						paronychia	75
						onychocryptosis	75
						other	75
Amputation			Yes/no	Move to deformity	100		
Footwear			Style	Type	100	Habits	86
						Worn always	100
						Outdoors only	100
						never	100
						Indoors only	100
Charcot			Visual assessment of redness, unilateral temp difference, swelling	Yes/no Move to deformity	100		

All of the proposed risk factors were marked ‘yes’ by the panelists in response to the question of practicality. Panelists marked 3 methods of recording risk factors as ‘No’ and 1 was marked as unclear. It was felt by the panelists that previous amputation should be recorded as a yes/no followed by the site and whether it was traumatic, surgical or auto amputation. It was further felt that the recording of skin and nail conditions should also be done by circling yes or no, and that location of lesions should be recorded on pictures of both a left and right foot. Other lesions to be recorded here include amputations and current ulcers. Smoking was marked as unclear by 6 (75 %) of the panelists as they felt that this was not specific enough due to the differing methods of tobacco use in India which include smoking, chewing and snuff (Table 7).

**Table 7:** Delphi results: Round 4-Pilot of the tool

Risk factors confirmed for inclusion in the Pilot tool	Practicality of proposed risk factor	% expert consensus	Tests and assessment methods confirmed for inclusion in the Pilot tool	Method of recording tests and assessment methods	Practicality of proposed test/assessment and method of recording	% expert consensus
Peripheral vascular assessment	Yes	100	DP/TP pulses palpable	Yes/no	Yes	100
			Temp touch	Normal/Abnormal	Yes	100
			Intermittent claudication	Yes/no	Yes	100
			Oedema Present	Yes/no	Yes	100
			Rest pain	Yes/no	Yes	100
Neurological assessment	Yes	100	Monofilament	10 sites Hypopigmented skin lesions	Yes	86
			Vibration	Left/Right Normal/Absent/reduced	Yes	100
Deformity	Yes	100	Charcot	Yes/no	Yes	100
			Amputation	Site and Surgical/traumatic/auto	No	75
			Forefoot	Specific toe/site Left and right	Yes	100
			Rear foot	Foot posture RCSP Normal/pronated/supinated	Yes	75
Skin conditions	Yes	100	Fungal	Tick box	No	75
			Cellulitis	State site		
			Current ulcer	Left or right foot		
			Previous ulcer	For Other state condition		
			Normal			
			Interdigital rash			
			Hyperhydrotic			
			Hypohydrotic			
			Fissure			
			Corns and Callus			
			Extravasation tissue			
			Blisters			
			Other			
Nail conditions	Yes	100	Onychauxis	Tick box State site	No	100
			Onychomycosis	Left or right foot		
			Paronychia	For Other state condition		
			Onychocryptosis			
			Other			
Smoking	Yes	100		Yes/no	Unclear	75
Footwear	Yes	100	Type	Tick box	Yes	100
				State Type		
			Habits	Worn always	Yes	100
				Outdoors only		
				Indoors only		
				Never		
Significant Surgical History	Yes	100		Location, Details	Yes	100

The panelists also suggested that a number of sections be added to the final assessment tool which had not been considered until the tool had been used in practice (Table 8).

**Table 8:** Delphi results: Round 4-Additional suggested items to be added to final tool

Proposed additions to the assessment tool	Further details
Social to be added to the demographics section	This will include smoking and additionally alcohol due to the high reported incidence of alcoholism by the panel members
Filarial to the medical history section	
Patient complains of (in relation to the foot condition)	To record patients chief complaint
Classification and stage of diabetic foot	To aid putting patients onto the correct management plan
Management plan	This would include
	Initial Return period
	Next screening
	Treatment (interventions to circle)
	Referral (departments to circle)
	As a quick reference to past treatments
Diagnosis/summary	A small section at the end of the tool to allow some notes to be written about the patients diagnosis

At this stage all eight of the panel members reported that they endorsed the Delphi procedure following a review of the aims and purpose. Additionally all eight panel members reported use of the evidence provided and the feedback from the previous rounds. Two members stated that although they had read the evidence initially they used the feedback predominantly to inform and support their decisions as the evidence document took time to read in what were already busy clinics for them to manage.

### Delphi round 5

All eight of the practitioners involved with the process agreed to being observed using the tool within their own departments.

At this stage the panel members used the tool and the researchers observed it in use for one week within the hospital. All panel members agreed on the alterations to the way in which amputations, skin and nail conditions were recorded via a yes/no structure and the recording of the sites of the skin and nail conditions onto pictures. No panel members recorded any items as ‘unclear’ (Table 9). Six of the items proposed for additional inclusion were accepted by all of the panel members and therefore added to the final tool. The item Diagnosis/summary however was felt by seven members of the panel (88 %) to be unnecessary. It was argued that as the tool collects multiple pieces of data it would also produce many possible diagnoses, which would be, prioritized differently depending on the specialist using the tool. It was further expressed that this was not required as the additional classification category provided enough information about risk, to guide the management steps appropriately. The diagnosis/summary section was therefore excluded from the final tool (Table 10).

**Table 9:** Delphi results: Round 5-Alterations to methods of recording

Risk factors confirmed for inclusion in the Pilot tool	Tests and assessment methods confirmed for inclusion in the Pilot tool	Method of recording tests and assessment methods	Practicality of proposed test/assessment and method of recording	Suggested alternative	% expert consensus	Comments or amendments on previous round
	Amputation	Site and Surgical/traumatic/auto	No	Yes/no + recorded on foot picture	100	Not challenged and therefore included
Skin conditions	Fungal	Tick box	No	Yes/no + recorded on foot picture	100	
		State site				
	Cellulitis	Left or right foot				
	Current ulcer	For Other state condition				
	Previous ulcer					
	Normal					
	Interdigital rash					
	Hyperhydrotic					
	Hypohydrotic					
	Fissure					
	Corns and Callus					
	Extravasation tissue					
	Blisters					
	Other					
Nail conditions	Onychauxis	Tick box	No	Yes/no + recorded on foot picture	100	
	Onychomycosis	State site				
	Paronychia	Left or right foot				
	Onychocryptosis	For Other state condition				
	Other					
Smoking		Yes/no	Unclear	Include smoking, snuff, Chewing	100

**Table 10:** Delphi results: Round 5-Additional items to be added to final tool

Proposed additions to the assessment tool	Further details	Acceptance of additional item	% expert consensus	Comments or amendments on previous round
Social to be added to the demographics section	This will include smoking and additionally alcohol due to the high reported incidence of alcoholism by the panel members	Yes	100	Not challenged and therefore included
Filarial to the medical history section		Yes	100	
Patient complains of (in relation to the foot condition)	To record patients chief complaint	Yes	100	
Classification and stage of diabetic foot	To aid putting patients onto the correct management plan	Yes	100	
Management plan	This would include	Yes	88	
	Initial Return period			
	Next screening			
	Treatment (interventions to circle)			
	Referral (departments to circle)			
	As a quick reference to past treatments			
Diagnosis/summary	A small section at the end of the tool to allow some notes to be written about the patients diagnosis	No	88	Not challenged and therefore excluded

The tool is structured as a list of thirteen sections made up of the risk factors identified during this investigation. Each of those sections contains subsections made up of the relevant tests, assessment methods and visual checks used to identify foot pathologies. The participants agreed that the following items (Table 11) were to be highlighted as ‘red flags’ and should therefore alert a rapid referral to the appropriate department. Participants requested that these items were highlighted on the tool with a capital letter R. The final tool is presented in Additional file 1. The clinical content of tool itself is not greatly dissimilar to foot health assessment tools developed in the West, this however is not surprising as the guidelines on which SIFT was based were mostly developed in the West. More importantly the opportunity for the clinicians to be participatory in the process has developed a tool where the structure and format are born out of the Indian health care system and subsequent adoption has taken place as a result of the participation in its development. A more detailed description of each section together with the corresponding evidence base were issued to the participants to support implementation and to act as a reference once the researchers had withdrawn from the location on completion. The guidelines and use of the tool in round 5 identified possible training needs for the participants and potentially for others who may also wish to use the tool at this site. Therefore in conjunction with the guidelines the researchers also developed and delivered a short foot health assessment and management training program entitled ‘Principles of lower limb assessment and management’ (Additional file 2). The SIFT was subsequently adopted by the hospital and used in practice. To date the tool has been used 2,622 times.

**Table 11:** Items to be highlighted as red flags in final tool

Medical conditions	Diabetes
	Leprosy
	Buergers disease
	PVD
	Venous insufficiency
	Rheumatoid arthritis/SLE
	Filarial
Skin conditions	Current Ulcer
	Previous Ulcer
	Cellulitis
Deformity	Charcot
	Previous amputation
PVD	IC
	Rest pain
Neurological system	Less than 8 sites
	Hypo pigmented lesions

## Discussion

Through the action research approach researchers facilitated the process whereby clinicians took local ownership of a clinical problem, developed their own solution i.e. the Salford Indian Foot health assessment Tool (SIFT) and embedded it into their practice. Through this approach researchers identified the knowledge and skills of the adopters, made the research evidence accessible and using Delphi ensured the adopters actively participated in reaching professional consensus on how that research evidence was embraced and embedded into practice [18]. This is the first time an action research approach has been used to drive change in Indian foot health care services

The Delphi process has been a useful method for both data collection and to achieve consensus with the participants [19, 20]. Delphi was employed as a tool embedded within the ethos of action research. Active participation of the adopters in this process has proven to be effective as a vehicle to embed evidence into practice, facilitate ownership of the solutions thereby sustaining change within their clinical practice [21].

It is generally considered that the Delphi technique requires ‘experts’ to be the participants, however, it can still be used where no experts exist [23]. In the west, podiatrists are considered the experts in the care of foot problems [3, 11] but in India there are no locally trained podiatrists within its healthcare system. However, although the participants were not experts in the area of foot health assessment they were experts in how foot health problems present in their clinics and in India on a daily basis. Critically, it was this expertise, not expertise in best practice of foot health, that was needed to drive creation of shared solutions for the foot assessment tool [22–25].

The Delphi has the further advantage of offering anonymity [26]. This helped prevent domination of some over others due to caste, gender, authority or personality. It is recognized that this culture and environment may influence the success of a change management process [27].

According to the institutional theory [27], the institutional environment can be defined by its structure and culture. Hofstede [28] identifies ‘power distance’ (PDI) as the extent to which the less powerful members of institutions accept and expect that power be distributed unequally, in this context it is the caste system [29]. Hofstede [28] also identified that gender inequality influences the culture of an institution and in India the male gender is viewed as superior with the role of decision makers [30, 31, 32]. Hence, we considered the anonymity of the Delphi method would overcome some of these influences in order to achieve completion of the action research cycle.

There are limitations to the use of the Delphi method [33]. There is no reported standard for the size and selection of panel members, with studies using a range of 4–171 experts, [34–36]. Further, there is no “typical” Delphi method, rather that the method is modified to suit the context and the research question. Additionally, there is no evidence of reliability of the method and as a result it cannot be certain that if the same task were given to two or more different panels, would the same results be obtained. Furthermore the lack of opportunity to clarify responses can create interpretation difficulties for both the researchers and participants [37], especially when working with participants whose first language is not English. The Delphi method has been used successfully to develop data collection tools previously [38–43] but to date, this is the first time that a delphi has been used within an action research approach to bring about local ownership of a clinical problem and produce a solution which was the development of the Salford Indian Foot health assessment.

However, the SIFT is not without its limitations and clearly focuses on the medical and podiatric presentations of patients following a biomedical model of health care. Although it collects some data in relation to alcohol, tobacco use and footwear practices, the wider bio psychosocial aspects [44, 45] are absent. It is important to handle the three together, as literature suggests that patient perceptions of health, threat of disease and barriers to good health in a patient's social or cultural environment influences the likelihood that they will engage in good health behaviors [46]. The dominance of the biomedical model may reflect wider cultural approaches to health care.

Use of the tool in clinical practice to assess consistency and construct validity are ongoing, as at this stage we have not undertaken any formal quantitative analysis of the SIFT. However, the first stages of the validation process have included a pilot of the tool in clinical practice. This has shown variation among some clinicians where there is subjectivity in the assessment decision which is similar to what Thompson et al. 2004 found. [47]. The pilot also identified that there was a training need for objective clinical testing. The use of ‘objective’ diagnostic equipment has been shown to reduce variability in the conduct of clinical tests [48–50]. It has also been reported that the opportunity to make modifications based upon reflections in practice can be useful in improving validity and reliability when generalists use structured screening tools [51]. Further testing is required to measure the reliability of the instrument and thereby determine the degree of consistency between the scores obtained at two or more independent times of testing by measuring inter rater reliability [52].

The participants reflected on the final tool and it components and this highlighted the need for training to support its use [53, 54]. Hence, as well as the tool being the vehicle for assessing foot health, it aided identification of training needs in foot health assessment and management including an increased awareness of the importance of simple lesions, the importance of regular screening including vascular, neurological, wound and offloading principles in the high risk foot. Further, the participants identified a need for sustainable training [55] and to meet this requirement a ‘train the trainer’ programme was delivered. The combination of the action research approach to develop the SIFT and the training programme aligned with the recommendation for multiple strategies to achieve improved knowledge and change in behavior made by Halfens and van Linge (2003) [6] and when combined with the education of the local ‘opinion leader’, both the need and the process of change was optimized [56, 57].

This research has facilitated the action research cycle that initially identified the need for change [5]. Through the phases of action and implementation the authors have facilitated the development of a locally defined, context specific assessment tool to aid identification of foot problems and hence the implementation of appropriate and timely management for individual patients. Further, the information gathered from this tool can be used to identify areas for service improvement.

## Conclusion

The action research process has given local ownership of the solution to practitioners and produced the first systematically developed evidence based foot health assessment tool to be used in India. The first step identified by the participating practitioners [5], as being pivotal to achieving better outcomes for patients. Engagement in the action research process has given practitioners the opportunity to reflect on current practice and bring about change within their service and individual clinical practice.

## Additional files

Additional file 1:
**Copy of Salford Indian Foot Health Assessment Tool (SIFT). **(DOC 170 kb) Additional file 2:
**Learning points for foot health assessment and management training.** (DOC 33 kb)
