# Supplementary material for: An action research approach to facilitating the adoption of a foot health assessment tool in India
Source: J Foot Ankle Res. 2015 Sep 16;8:52. doi: 10.1186/s13047-015-0108-3 (PMC4574208; doi:10.1186/s13047-015-0108-3)
Supplement: Additional file 1: — Copy of Salford Indian Foot Health Assessment Tool (SIFT). (DOC 170 kb) [file 13047_2015_108_MOESM1_ESM.doc]

**SRU Foot Clinic**

**Foot Assessment**

**Date of assessment………………… Hosp/op/reg.no………………...**

**Patient demographics**

| **Name** |  | | | |
| --- | --- | --- | --- | --- |
| **Date of Birth** |  | **Age** |  | |
| **Sex** (circle) | **Male** | **Female** | | |
| **Tel/Mobile no** |  | | | |
| **Occupation** |  | | | |
| **Social** (circle) | **Smoking / Chewing Tobacco / Snuff** | | | **Alcohol** |
| **Referred From** |  | | | |

**Medical history**

| **Condition** | **Tick**  **if yes** | **Medication/treatment to date** | **Circle** |
| --- | --- | --- | --- |
| Diabetes |  |  | **R** |
| Leprosy |  |  | **R** |
| Buergers disease |  |  | **R** |
| PVD |  |  | **R** |
| Venous insufficiency |  |  | **R** |
| Rheumatoid arthritis/SLE |  |  | **R** |
| Filarial |  |  | **R** |
| Musckuloskeletal |  |  |  |
| Other |  |  | **R** |

**Significant Surgical History**

| **Location** | **Details** |
| --- | --- |
|  |  |
|  |  |
|  |  |

**Foot Pathology**

Patient complains of …………………………………………………………………………… ………………………………………………………………………………………………….………………………………………………………………………………………………….………………………………………………………………………………………………….………………………………………………………………………………………………..……………………………………………………………………………………………….……………………………………………………………..…………………………………………

**On Examination**

**Nail conditions (Refer to picture for location)**

| **Tick here** | **On Examination** | **Circle** | |
| --- | --- | --- | --- |
|  | Onychauxis | Yes | No |
|  | Onychogryphosis | Yes | No |
|  | Onychomycosis | Yes | No |
|  | Paronychia * | Yes | No |
|  | Onychocryptosis* | Yes | No |
|  | Other  Condition (state) | Yes | No |

**Skin condition (Refer to picture for location)**

| **Tick**  **here** | **On examination** | **Circle** | |
| --- | --- | --- | --- |
|  | Normal | Yes | No |
|  | Callus | Yes | No |
|  | Corns | Yes | No |
|  | Above + extravasation * | Yes | No |
|  | Blister* | Yes | No |
|  | Fissure | Yes | No |
|  | Interdigital rash | Yes | No |
|  | Hypohydrotic | Yes | No |
|  | Hyperhydrotic | Yes | No |
|  | Above + open * | Yes | No |
|  | Current Ulcer R | Yes | No |
|  | Previous Ulcer R | Yes / No |  |
|  | Cellulitis R | Yes / No |  |
|  | Other |  |  |

**Footwear**

| **Tick**  **here** |  | **Type**  **e.g shoes ,sandals, or chappals** |
| --- | --- | --- |
|  | Worn always |  |
|  | Indoors only |  |
|  | Outdoors only |  |
|  | Never R | |

**Deformity assessment**

| **forefoot** | **Specific toe deformity** | **Site (Left)** | **Site (Right)** |
| --- | --- | --- | --- |
|  |  |  |
|  |  |  |
|  |  |  |

| **rearfoot** |  | **Right** | **Left** |
| --- | --- | --- | --- |
|  | Foot posture RCSP | Normal / Pronated / Supinated | Normal / Pronated / Supinated |
| **other** | Charcot foot R | Yes / No | Yes / No |
|  | Previous amputation  Site of amputation  Type R | Yes / No  ………………………………………..  Surgical / Traumatic / Autoamputation | Yes / No  ………………………………………….  Surgical / Traumatic / Autoamputation |

(Circle where appropriate)

**Add position of all lesions to pictures**

**RIGHT LEFT**

**
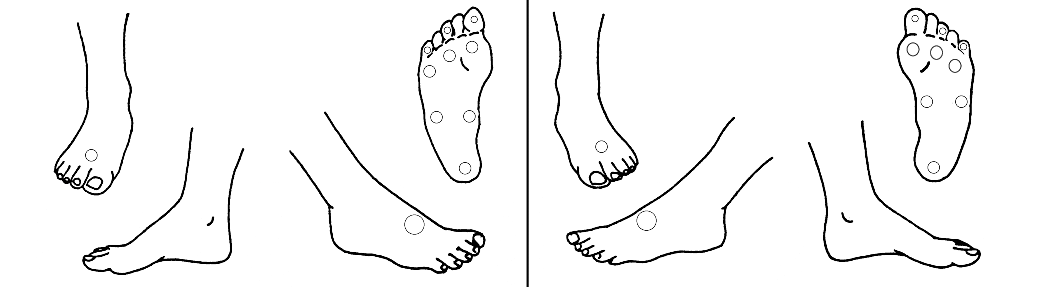
**

**Peripheral Vascular System**

|  | Left | Right |
| --- | --- | --- |
| Dorsalis pedis palpable | Yes / No | Yes / No |
| Posterior tibialis palpable | Yes / No | Yes / No |
| Intermittent claudication R | Yes / No | Yes / No |
| Rest Pain R | Yes / No | Yes / No |
| Temperature | Normal / Abnormal * | Normal / Abnormal * |

Presence of Oedema Yes / No

**Neurological Assessment**

| Right | | | Left | | |
| --- | --- | --- | --- | --- | --- |
| Normal | Absent | Reduced | Normal | Absent | Reduced |

Vibration hallux

| **10g Monofilament** | Right | | Left | |
| --- | --- | --- | --- | --- |
| Plantar hallux | Present | Absent | Present | Absent |
| Plantar 1^st^  met head | Present | Absent | Present | Absent |
| Pulp of 5^th^ toe | Present | Absent | Present | Absent |
| Plantar 5^th^ met head | Present | Absent | Present | Absent |
| Plantar styloid process | Present | Absent | Present | Absent |

**Score out of 10 sites; ………………….. If less than 8 R**

**NB - In addition to 10 sites all HYPOPIGMENTED SKIN LESIONS should be tested and areas of anaesthesia noted here. R ……………………………………..**

**…………………………………………………………………………………………**

**Diagnosis**

**..............................................................................................................................................................................................................................................................................................................................................................................................................................**

**Classification and Stage (**Circle as appropriate)

| **Normal** | **Ischaemic** | **Neuropathic** | **Neuroischaemic** |
| --- | --- | --- | --- |

| **Stage** | **Circle foot type** |
| --- | --- |
| **1** | **Normal** |
| **2** | **High risk** |
| **3** | **Ulcerated** |
| **4** | **Infected** |
| **5** | **Necrotic** |
| **6** | **Unsalvageable** |

**Management Plan**

| **Initial Return period** |  | | | | | | |
| --- | --- | --- | --- | --- | --- | --- | --- |
| **Next screening** |  | | | | | | |
| **Treatment**  **(circle)** | Nails | Debridement | Enucleation | Pressure relief | Casting | Orthotics | Strapping |
|  | Stretching | Medicines | Education | Dressings | Ultrasound | Other therapy | |
| **Referral**  **(circle)** | Orthopaedics | Diabetes and endocrinology | Vascular | General surgery | Dermatology | Orthotist | Physiotherapy |
