# Supplementary material for: An action research approach to facilitating the adoption of a foot health assessment tool in India
Source: J Foot Ankle Res. 2015 Sep 16;8:52. doi: 10.1186/s13047-015-0108-3 (PMC4574208; doi:10.1186/s13047-015-0108-3)
Supplement: Additional file 2: — Learning points for foot health assessment and management training. (DOC 33 kb) [file 13047_2015_108_MOESM2_ESM.doc]

**Additional file 3**

**Learning Points for foot health assessment and management training**

| **Topic** | **Outline** |
| --- | --- |
| Infection control | - Hand hygiene - Use of PPE - Preparation of the clinical environment - Procedures when undertaking routine podiatry - Safe use and disposal of sharps - No touch techniques. (also see wound cleansing) |
| Basic skin lesions | - Overview of basic skin lesions to include differential diagnosis and management - Callus debridement and corn enucleation through alginate/ model foot practice followed by supervised patient management in university clinic |
| Vascular and neurological assessment | - Overview of basic vascular assessment - Pulses, colour, temperature, Doppler - ABPI - Overview of basic neurological assessment - Monofilament and VPT - Neuropathic disability Score (NDS) |
| Biomechanics and pressure relief | - Overview of lower limb motion. - Overview of the kinematic and kinetic parameters of normal gait and principles of joint examination in the lower limb. - Overview of foot types, deformity, foot pathology and causes of abnormal pressure - Principles of pressure relief and techniques for successful correction. |
| Wound assessment and management | - Wound identification and classification - Wound cleansing - Wound debridement - Wound and exudate management - Off loading ( briefly) - Identifying infection |
